# Anisotropic δ-to-α Phase Transition in Formamidinium Lead Iodide Thin Films

**DOI:** 10.1021/acsnano.5c00037

**Published:** 2025-02-25

**Authors:** Chen Yang, Changsheng Chen, Tieyuan Bian, Chao Xu, Xiangli Che, Dongyang Li, Kuan Liang, Xuezhe Dong, Jun Yin, Gang Li, Ye Zhu

**Affiliations:** † Department of Applied Physics, Research Institute for Smart Energy, The Hong Kong Polytechnic University, Hung Hom, Kowloon 00000, Hong Kong, China; ‡ Department of Applied Physics, The Hong Kong Polytechnic University, Hung Hom, Kowloon 310028, Hong Kong, China; § Department of Electrical and Electronic Engineering, Research Institute for Smart Energy, Photonic Research Institute, The Hong Kong Polytechnic University, Hung Hom, Kowloon 310028, Hong Kong, China

**Keywords:** 4D-STEM, hybrid perovskites, FAPbI_3_, polarized light microscopy, phase transition

## Abstract

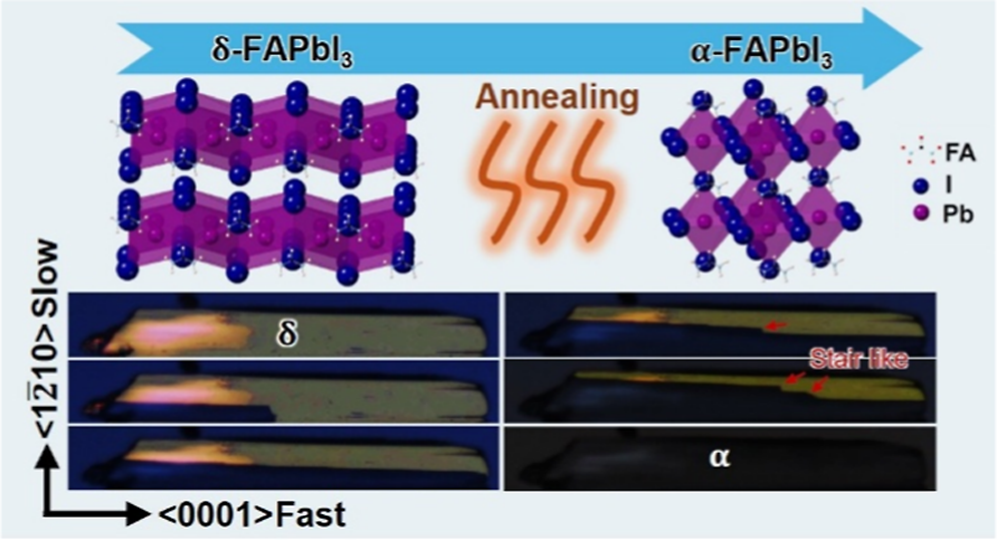

Thermal annealing on hybrid perovskites is essential to prepare high-quality solar cells with extraordinary efficiency, whose benefits include transformation of inactive phases such as δ-FAPbI_3_ to active α-FAPbI_3_. The detailed mechanism for such critical phase transition, however, has not yet been adequately studied. Here, we present multiscale microscopic observation to unravel the anisotropic δ-to-α transition in epitaxial FAPbI_3_ thin films. We adopt polarized light microscopy that offers enhanced contrast to distinguish isotropic α-FAPbI_3_ from the anisotropic δ-FAPbI_3_. Facilitated by in situ heating, it allows us to identify heterogeneous nucleation of α-FAPbI_3_ and the subsequent diffusional phase transition preferentially occurring along ⟨0001⟩, which is underpinned by the smaller activation energy along the face-sharing direction of PbI_6_ octahedra. We further reveal the morphology and orientation relationship at the δ-to-α transition front using four-dimensional scanning transmission electron microscopy (4D-STEM), evincing the surface energy dominated orientation rather than the interfacial energy. The presence of high-density planar defects is also discovered at the transition front, which can be considered as an intermediate state facilitating δ-to-α structure transformation. Besides filling the knowledge gap on the phase transition behavior in FAPbI_3_, our work also demonstrates a multiscale microscopy approach to interrogate the phase transition mechanism in hybrid perovskites.

## Introduction

Hybrid perovskites based on formamidinium lead iodide (FAPbI_3_) have been recognized as one of the most promising candidates for next-generation solar cells with exceptionally high efficiency and lower cost. Owing to the instability of the cubic perovskite structure inherited from the large ionic radius of NH_2_CH=NH_2_^+^ (FA^+^), at room temperature, FAPbI_3_ tends to form a hexagonal nonperovskite structure (δ-FAPbI_3_ or yellow phase)^1^ without photoactivity. Thermal annealing is usually required to induce phase transition and to achieve the photoactive cubic phase (α-FAPbI_3_ or black phase),^2^ which can be maintained down to room temperature for solar cell applications. A plethora of works have been demonstrated to prepare high-quality α-FAPbI_3_ materials with record-high power conversion efficiencies based on such annealing-induced δ-to-α phase transition.^3−10^ Due to its critical impact on solar cell performance, significant efforts have been made to optimize the annealing process. For example, radiative-assisted thermal annealing has been developed to achieve a more efficient phase transition, which can obtain purer α-FAPbI_3_ with less annealing time.^11^ Atmosphere control during annealing has also been established as an effective approach to lower the δ-to-α transition barrier and to improve crystal quality of α-FAPbI_3_.^6,12^ Moreover, higher δ-FAPbI_3_ crystallinity prior to annealing can promote the formation of α-FAPbI_3_ with larger grains and more aligned orientation, leading to enhanced performance of FAPbI_3_-based solar cells.^8,9^

Despite its vital role in the quality and performance of solar cells, however, the fundamental mechanism of the δ-to-α phase transition in FAPbI_3_ remains unclear. Sánchez et al. examined the impact of heating rate on δ-to-α transition in polycrystalline FAPbI_3_ films using X-ray diffraction (XRD) and differential scanning calorimetry but without microscopic observation to reveal the phase transition process.^13^ Lai et al.^20^ have applied pioneering in situ optical microscopy on the δ-to-α phase transition in FAPbI_3_, showing its lower activation energy than inorganic perovskites. Nevertheless, the observation was limited to one-dimensional (1D) FAPbI_3_ microwires only, which is very different from FAPbI_3_ films used in solar cells. The limited resolution of optical microscopy is also incapable of revealing the detailed structure and morphology at the transition front, leaving a significant knowledge gap hampering our understanding of the phase transition mechanism. In this work, we demonstrate multiscale microscopic observation on δ-to-α phase transition in FAPbI_3_ thin films that are more relevant to solar cell devices. In particular, we prepare epitaxial δ-FAPbI_3_ thin films with a well-defined orientation for this mechanistic study. To better detect the phase transition process, we adopted polarized light microscopy (PLM) that offers enhanced contrast between isotropic α-FAPbI_3_ and anisotropic δ-FAPbI_3_. Facilitated by in situ heating, it enables the observation of heterogeneous nucleation of α-FAPbI_3_ and the subsequent anisotropic δ-to-α phase transition underpinned by the lower kinetic energy barrier along ⟨0001⟩. We further reveal the morphology and orientation relationship at the δ-to-α transition front using 4D scanning transmission electron microscopy (4D-STEM). The presence of high-density planar defects is also unveiled at the transition front, which may promote a structural transformation between δ- and α-FAPbI_3_.

## Results and Discussion

**Figure 1:**
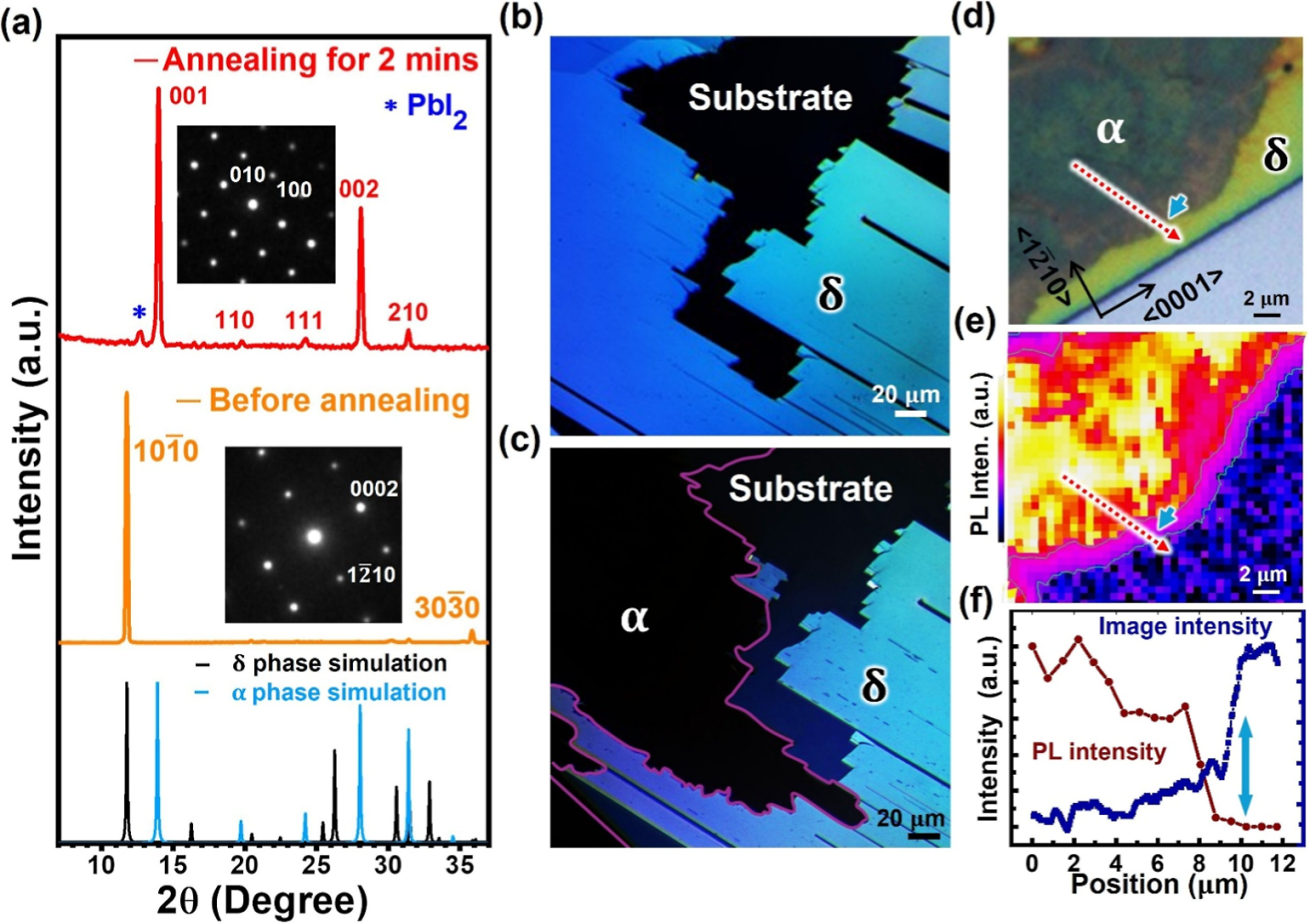
(a) Ex situ XRD of a FAPbI_3_ thin film before (yellow) and after (red) annealing at 160 °C, showing δ-to-α phase transition as indicated by the reference XRD at the bottom. Insets: electron diffraction patterns taken from corresponding samples. (b,c) PLM on a FAPbI_3_ thin film (b) before and (c) after annealing, showing δ-to-α phase transition at the upper-right corner. (d–f) Confocal PL mapping on a δ-to-α transition front with the (d) optical image, (e) PL intensity map from the same region, and (f) PL (brown) and image intensity (blue) profiles along the red dotted arrows in (e) and (d), respectively, with the position of the boundary indicated by the cyan arrow.

We prepare epitaxial δ-FAPbI_3_ thin films as large as a few millimeters, using the antisolvent-assisted space-confined method at ambient temperature (see details in Methods and Figure S1).^14^ A uniform thickness of 50–100 nm can be achieved with only a few nanometers of surface roughness as measured by profilometry and atomic force microscopy (Figure S2). XRD evinces the high-purity δ-phase with a single-crystal structure (*P*6_3_/*mmc*, #194)^2^ oriented along the {101̅0} plane normal (yellow curve in Figure 1a), which has been verified by electron diffraction in the inset. The high crystalline quality of the films is further evidenced by rocking curve measurement (Figure S2d), showing the narrow peak width comparable to bulk single crystals grown by the inverse temperature crystallization approach.^15,16^ Such large-scale single-crystal thin films can be transferred onto TEM grids, allowing the investigation of the phase transition behavior using both optical and electron microscopy at the multiscale.

**Figure 2:**
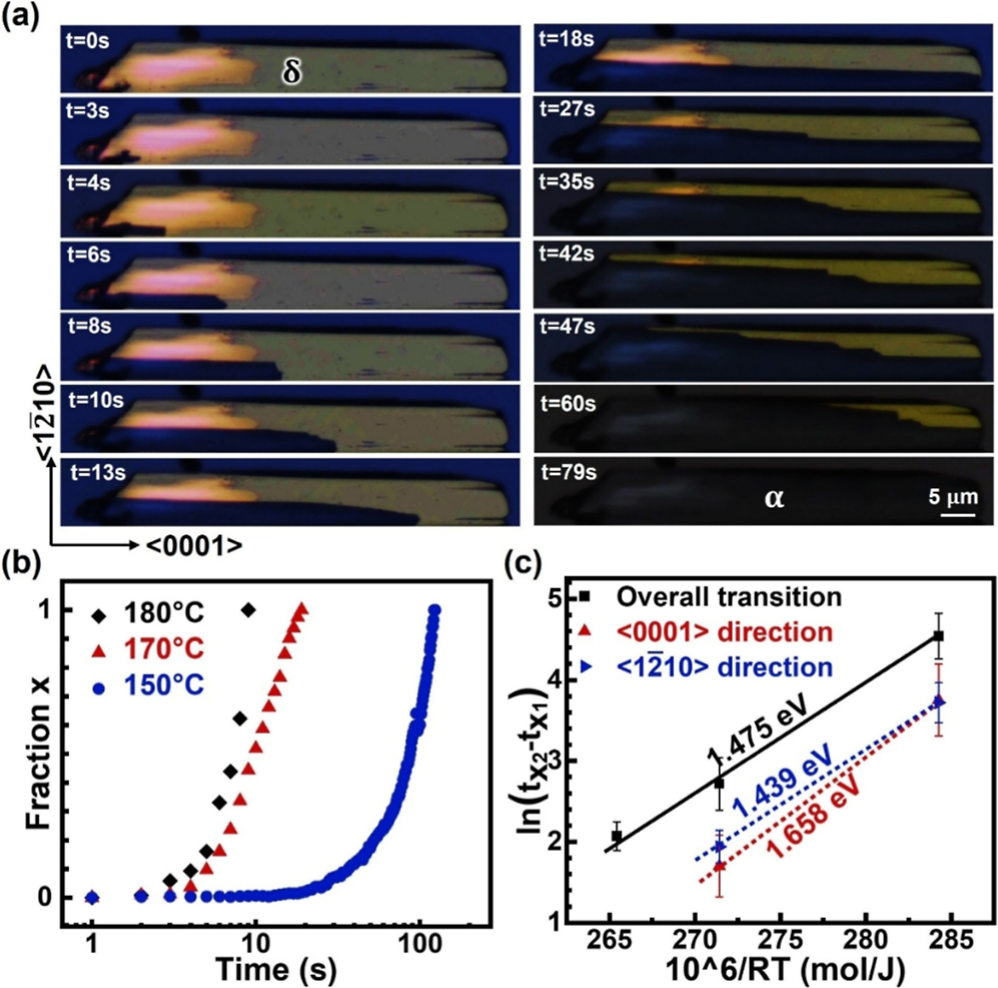
(a) Time-series PLM images showing δ-to-α phase transition induced by in situ annealing at 170 °C. (b) Measured α-FAPbI_3_ area fraction as functions of annealing time at 150, 170, and 180 °C, respectively. (c) Derivation of activation energies for δ-to-α phase transition along ⟨0001⟩ and ⟨12̅10⟩ based on the JMA model (see Note S1).

By annealing the δ-FAPbI_3_ films in a N_2_ atmosphere, δ-to-α phase transition occurs as indicated by the dominant α-FAPbI_3_ (*Pm*3̅m, #221)^17^ signal from XRD (red curve in Figure 1a). With the isotropic structure, α-FAPbI_3_ appears dark under PLM, which can be distinguished from the anisotropic δ-FAPbI_3_ that appears bright (Figures 1c and S3). This contrast interpretation has been explicitly confirmed by photoluminescence (PL): the dark area exhibits the characteristic α-FAPbI_3_ signal at ∼800 nm, whose intensity diminishes across the boundary toward the bright δ-FAPbI_3_ regions (Figures 1e,f and S4). Compared to the normal optical image in Figure 1d, PLM offers substantially enhanced contrast that can be utilized to track the phase transition dynamics by using in situ heating. As shown in Figure 2a (also see the in situ movie in Supporting Information), the dark α-FAPbI_3_ phase nucleates at the lower-left corner, reflecting heterogeneous nucleation, and preferentially propagates along the ⟨0001⟩ direction. The stark contrast between the pristine and transformed regions suggests that phase transition happens through the whole thickness along the ⟨101̅0⟩ direction, forming sharp boundaries between the two regions without overlapping (also seen in Figure 1c). The dark α-FAPbI_3_ regions propagate continuously and gradually, indicating that δ-to-α phase transition is achieved through atom diffusion (or diffusional transformation) instead of collective structure transformation (or displacive transformation).^18^ Using the fraction of the α-FAPbI_3_ area to indicate the transition progress, it consistently exhibits the exponential increase with time at various temperatures, with faster increase at higher temperature as shown in Figure 2b. This transition behavior can be described by the Johnson–Mehl–Avrami (JMA) model^19^
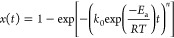
1where *E*_a_ represents the activation energy, *R* is the gas constant, *n* is the growth exponent, *k*_0_ is the rate constant prefactor, and *x* corresponds to the transition state represented by the α-FAPbI_3_ area fraction. The three curves measured at different temperatures in Figure 2b enable the derivation of *E*_a_ for the δ-to-α transition to be ∼1.475 eV (see Note S1). We note that this *E*_a_ value is comparable to the reported activation energy for polycrystalline FAPbI_3_ thin films (∼1.814 eV)^13^ but higher than what has been reported for 1D FAPbI_3_ microwires (∼0.84 eV).^20^ Despite the dimension difference, the latter work also used the simple fitting with an Arrhenius function, which is different from the dedicated JMA model adopted here for diffusional transformation.^21^

The observed δ-to-α transition in Figure 2a is dominated by ⟨0001⟩ propagation, consistent with the work by Lai et al. that demonstrated the preferential growth of δ-FAPbI_3_ nanowires and the preferential δ-to-α phase transition both along ⟨0001⟩.^20^ The measured average propagation rate along ⟨0001⟩ is ∼3.18 μm/s which is also remarkably close to their measured rate (∼3 μm/s at 163 °C) on 1D microwires. Such preferential phase propagation has been attributed to the face-sharing PbI_6_ octahedra along ⟨0001⟩ in δ-FAPbI_3_, which should transform into corner-sharing octahedra during δ-to-α transition. On the other hand, due to the limited dimension of their microwires, only 1D phase transition behavior was studied.^20^ In contrast, our FAPbI_3_ thin films allow us to investigate the anisotropic phase transition along different directions, such as the much slower propagation of α-FAPbI_3_ along the perpendicular ⟨12̅10⟩ direction (∼0.22 μm/s). The difference in the propagation speeds leads to a stair-like transition front with shorter steps along ⟨12̅10⟩, as observed at the late stage of transition (Figure 2a). Beam effect analysis on our δ-FAPbI_3_ films also reveals higher stability of {12̅10} stacking ordering than {0002} (see Note S2). The relatively unstable {0002} stacking may facilitate the phase transition to α-FAPbI_3_, resulting in the preferential propagation of the transition front along ⟨0001⟩. To better understand such anisotropic transition behavior, we further measure the area propagation rates along both ⟨0001⟩ and ⟨12̅10⟩ directions and derive the corresponding *E*_a_ values based on the JMA model. As shown in Figure 2c, *E*_a_ is ∼1.439 eV along ⟨0001⟩ and ∼1.658 eV along ⟨12̅10⟩, explaining the preferential phase transition along ⟨0001⟩. To the best of our knowledge, this is the first measurement of anisotropic activation energies for δ-to-α FAPbI_3_ phase transition.

**Figure 3:**
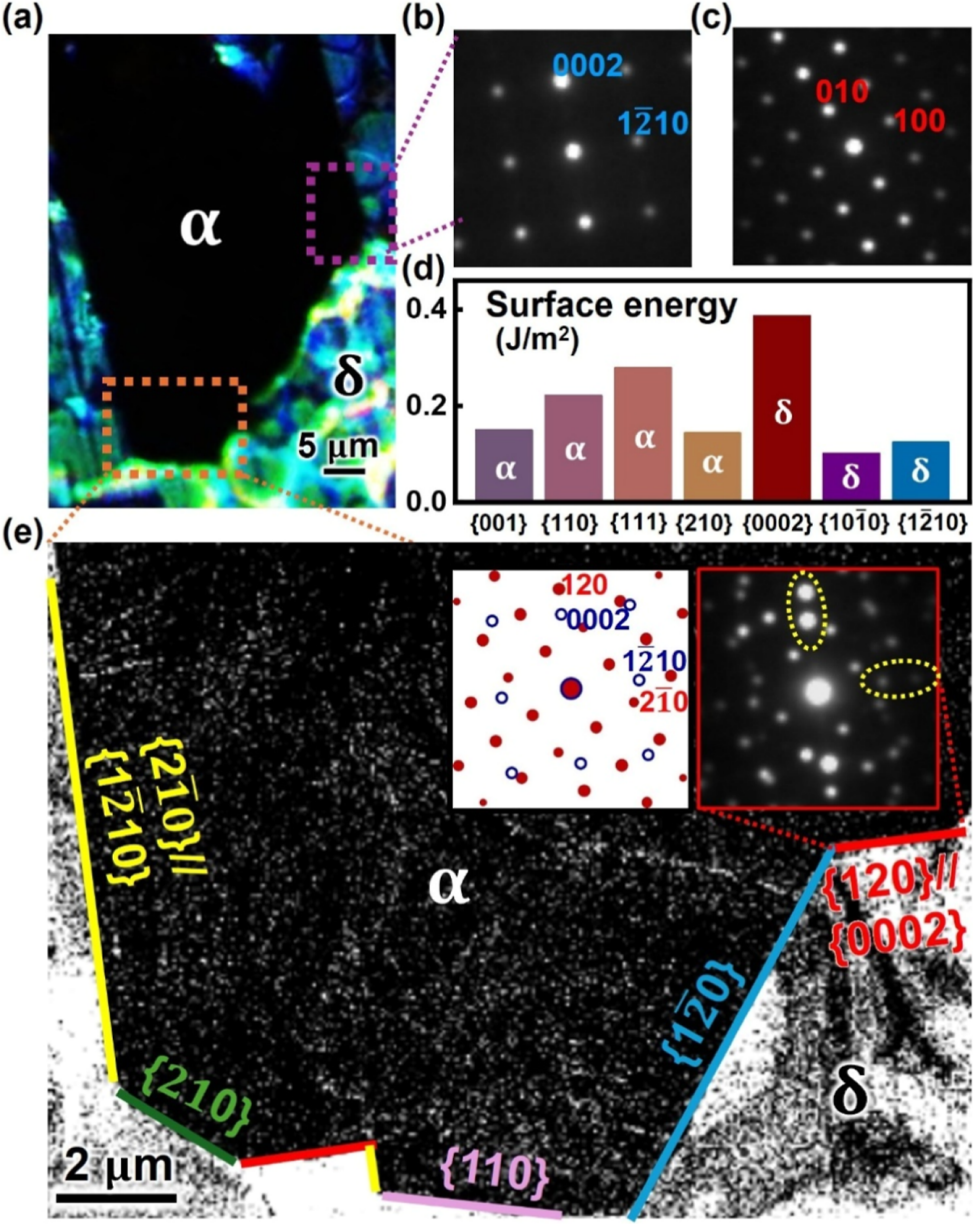
(a) PLM image showing a transition front between the colored δ-FAPbI_3_ and black α-FAPbI_3_. (b,c) Diffraction patterns from 4D-STEM showing (b) ⟨101̅0⟩ and (c) ⟨001⟩ zone axes for α- and δ-FAPbI_3_, respectively. (d) Density functional theory (DFT) calculation on surface energies of α- and δ-FAPbI_3_. (e) Dark-field image reconstructed using 0002 diffraction, showing the morphology of the transition front with the orientation relationship labeled in different colors. The upper-right inset is a diffraction pattern across the red boundary, showing the overlap of two phases with ⟨101̅0⟩_δ_//⟨001⟩_α_, {12̅10}_δ_//{210}_α_, and {0002}_δ_//{120}_α_, as illustrated by the schematic on the left.

It is noted that XRD in Figure 1a reveals the preferential orientation of transformed α-FAPbI_3_ along the {001} plane normal. It indicates the orientation relationship of ⟨101̅0⟩δ//⟨001⟩α, which does not follow the typical basal-type hexagonal-to-cubic transition (⟨0002⟩_H_//⟨111⟩_C_, {12̅10}_H_//{110}_C_, and {101̅0}_H_//{211}_C_) or the prismatic-type transition (⟨0001⟩_H_//⟨001⟩_C_ and {101̅0}_H_//{11̅0}_C_).^22−24^ We further explore the orientation relationship using 4D-STEM with spatially resolved diffraction across the transition front between δ and α phases. A special low-dose condition is used with a minimized beam effect on FAPbI_3_ (see Methods). Figure 3e is the dark-field image generated using 0002 diffraction, which shows dark α-FAPbI_3_ on top and bright δ-FAPbI_3_ at the bottom. δ-FAPbI_3_ is oriented along ⟨101̅0⟩ (Figure 3b), while α-FAPbI_3_ is oriented along ⟨001⟩ (Figure 3c), consistent with the orientations detected by XRD. The two phases are well-separated by the sharp transition front, with no overlap identified on either side, confirming the complete phase transition through the whole thickness (also see Figure S5). The longest transition front is along {12̅10}_δ_ in parallel with {210}_α_ (yellow lines in Figure 3a), consistent with the preferential propagation along the ⟨0001⟩_δ_ direction. Perpendicular to ⟨0001⟩_δ_, the transition front consists of a few segments with various orientations. Some segments show a well-defined orientation relationship (i.e., {0002}_δ_//{120}_α_ for the red lines), while others do not (blue and cyan lines in Figure 3a), showing rather complicated transition behavior between δ- and α-FAPbI_3_. Interestingly, despite the orientation variations, the majority of the transition front is aligned with {210}_α_, suggesting that they are habit planes of α-FAPbI_3_ with lower δ/α interfacial energy. Due to the susceptible nature of FAPbI_3_, we cannot achieve atomic-resolution imaging at the transition front to reveal the interfacial structure. On the other hand, even for the rational interfaces with ⟨101̅0⟩_δ_//⟨001⟩_α_, {12̅10}_δ_//{210}_α_, and {0002}_δ_//{120}_α_, we cannot find the way to form coherent interfaces between δ- and α-FAPbI_3_.

The incoherent δ/α-FAPbI_3_ interfaces have been proposed by Lai et al., who observed the same orientation relationship of ⟨101̅0⟩_δ_//⟨001⟩_α_ as our work. Howe et al. have considered the incoherent interfaces to be like high-angle grain boundaries, which may form liquid-like interfaces at high temperature.^25^ The liquid-like interfaces have been shown to facilitate the ion diffusion and the underpinned phase transition, as demonstrated by molecular dynamics simulations on CsPbBr_3_.^26^ Such incoherent interfaces also imply rather low interfacial energy between δ- and α-FAPbI_3_, so that the transition behavior should be dominated by the surface energy that leads to {001}-terminated α-FAPbI_3._^27^ The preferred {001} termination for α-FAPbI_3_ has been reported by many works,^28−31^ indicating its lower surface energy. This has been explicitly verified by DFT calculations showing lower surface energy for both {001} and {210} planes (Figure 3d), which explains the observed ⟨001⟩-oriented α-FAPbI_3_ with {210}-dominant habit planes at the transition front.

**Figure 4:**
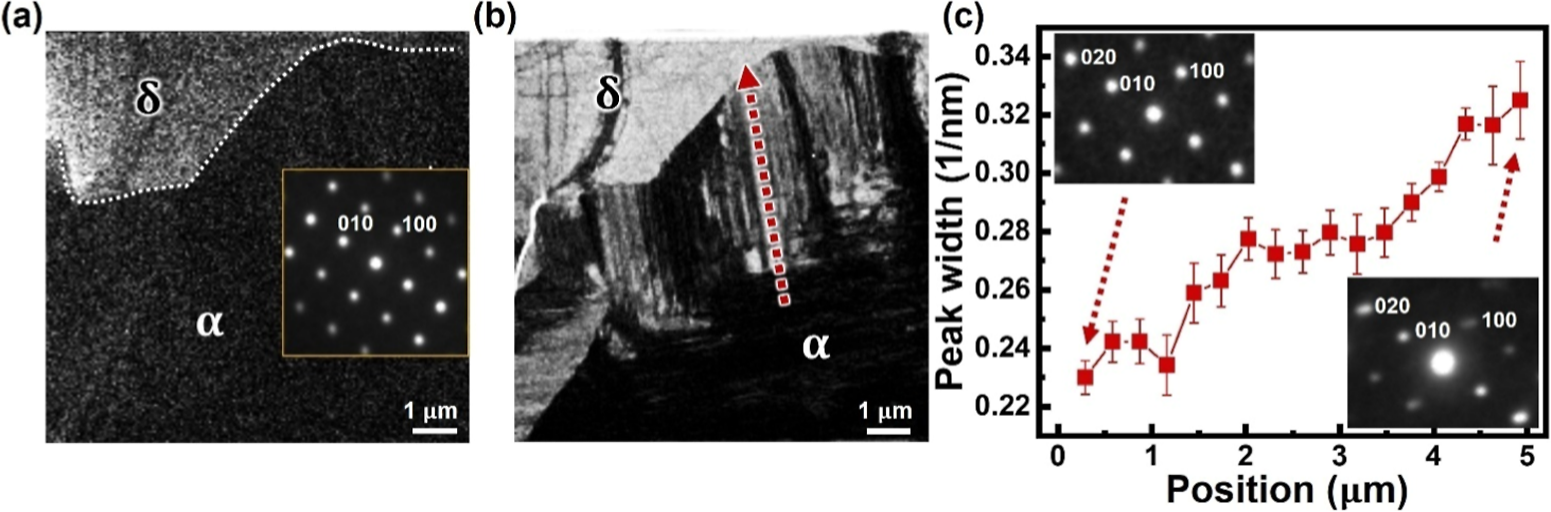
(a) Dark-field image reconstructed using 0002 diffraction, showing the δ-to-α transition front along the dotted line. (b) Reconstructed bright-field image from the same region as (a), showing high-density planar defects in the newly formed α-FAPbI_3_ near the transition front. (c) Measured 020 diffraction peak width along the red arrow in (b), showing increased defect density toward the transition front. The initial and final diffraction patterns are shown in the insets.

Careful inspection of the PL map in Figure 1d–f reveals a decrease of PL intensity inside α-FAPbI_3_ near the transition front, which suggests the presence of defects acting as the nonradiative recombination centers. This has been explicitly confirmed by 4D-STEM at the transition front: as shown by the reconstructed bright-field image in Figure 4b, α-FAPbI_3_ adjacent to the transition front exhibits high-density dark lines along ⟨110⟩, corresponding to planar defects such as stacking faults and twin boundaries. Planar defects have been identified along {111} in α-FAPbI_3_,^32,33^ whose intersection with {001} is indeed along ⟨110⟩,^34^ consistent with the observed dark-line contrast in Figure 4b. Such high-density planar defects break the long-range order perpendicular to the line direction, leading to the broadening of diffraction peaks along ⟨11̅0⟩ that has been observed in 4D-STEM diffraction patterns (lower-right inset of Figure 4c). Using the width of 020 diffraction along ⟨11̅0⟩ to reflect the defect density (or the degree of disorder), Figure 4c clearly unveils an increase of defect density in α-FAPbI_3_ toward the transition front. Since planar defects especially stacking faults have been identified as the deep recombination centers in α-FAPbI_3_,^32^ their increased density explains the reduced PL intensity near the transition front.

Observation in Figure 4 evinces that initially formed α-FAPbI_3_ near the transition front is highly defective with lower crystallinity. In particular, {111} planar defects such as stacking faults and twin boundaries change the local {111} stacking from A–B–C–A–B–C (cubic) to A–B–A–B (hexagonal) and can be considered as the intergrowth of δ phases within α-FAPbI_3_. It is thus not a surprise to see such high-density planar defects near the transition front, which may serve as an intermediate state that is typically observed during the basal-type hexagonal-to-cubic transition.^35−37^ On the other hand, with the observed orientation relationship distinct from the basal-type transition, such planar-defect-facilitated phase transition should be accompanied by the reorientation or even recrystallization process, which reorients the formed α-FAPbI_3_ to ⟨001⟩ with minimized surface energy. With further annealing, the initially formed defective α-FAPbI_3_ will develop better crystallinity with reduced defect density, as reflected by a much narrower diffraction peak width farther away from the transition front (Figure 4c), while the defective transition front further propagates into δ-FAPbI_3_ to continue the phase transformation.

## Conclusions

In summary, we have combined multiscale microscopy with in situ heating to directly observe the anisotropic behavior of the δ-to-α phase transition in FAPbI_3_ thin films. Our findings show that α-FAPbI_3_ nucleates heterogeneously upon heating, followed by a diffusional transformation that preferentially propagates along the ⟨0001⟩ direction, which is underpinned by the lower activation energy along the face-sharing direction of PbI_6_ octahedra. 4D-STEM further unveils the morphology and orientation relationship at the δ-to-α transition front, indicating the incoherent δ/α interfaces with potentially lower interface energy. The orientation of the transformed α-FAPbI_3_ is thus determined by the surface energy that favors ⟨001⟩-oriented α-FAPbI_3_. We have also identified high-density planar defects in newly formed α-FAPbI_3_ near the transition front, which appear to be an intermediate state promoting the δ-to-α transition. Our work not only deepens the understanding of the δ-to-α phase transition mechanism in FAPbI_3_ but also demonstrates a powerful approach combining in situ PLM and 4D-STEM to probe various phase changes in hybrid perovskites at a multiscale.

## Methods

### Epitaxial δ-FAPbI_3_ Thin Film Preparation

As illustrated in Figure S1, 1.6 M formamidinium iodide (Advanced Election) and lead iodide (PbI_2_, Advanced Election) were dissolved in γ-butyrolactone (Sigma-Aldrich) with thorough stirring, followed by filtration through a 2 mm filter to achieve a supersaturated precursor solution. A 3 μL aliquot of the precursor solution was carefully introduced into the interstitial space formed by aligning two octadecyltrichlorosilane (Sigma-Aldrich)-treated Si/SiO_2_ slices in a face-to-face configuration. The assembly was left undisturbed for 10 min to allow homogeneous precursor diffusion across the substrate interface via capillary action. The assembled slices were subjected to a self-designed pressure setup and sealed with chlorobenzene (CB, Sigma-Aldrich) as an antisolvent. The crystal growth process was conducted over 7 days, after which the slices were separated using a knife. All procedures were performed in a N_2_-filled glovebox to avoid exposure to H_2_O and O_2_.

### TEM Sample Preparation

Polystyrene (PS, Mw ∼ 35,000, Sigma-Aldrich) was dissolved in CB at 10 mg/mL. A 30 μL aliquot was deposited onto the thin film and spin-coated at 600 rpm. After drying in a glovebox for 30 min, the PS layer was carefully lifted with tweezers, transferred onto a legacy TEM grid, and rinsed with CB to remove residual PS.

### Material Characterizations

XRD was performed on a Rigaku SmartLab (9 kW, λ ∼ 1.54 Å). Optical alignment and sample height adjustments were performed to correct the omega offset and enhance signal contrast. The rocking curve of δ-FAPbI_3_ was measured at 2θ = 11.78° using the 2θ-omega scan method. The as-synthesized single-crystal thin films were encapsulated in an in situ annealing setup designed for optical observation and transferred from the glovebox to a Leica DM2700 M microscope. The microscope was equipped with a polarizer and analyzer set in a perpendicular configuration. Video data were recorded at a rate of 1 frame per second, while the temperature was uniformly increased at 50 °C per minute, ensuring precise control of thermal conditions throughout the experiment. A WITec alpha 300R instrument equipped with an Ar ion laser (∼532 nm) as the excitation source was used for confocal PL measurements, with the laser power reduced to 0.06 mW. A large-scale scan was performed over a region of tens of square micrometers with a resolution of 40 × 40 pixels. The sample was sealed to prevent humidity and oxygen exposure during the measurements. The 4D-STEM data sets were acquired using an EMPAD on a Thermo Fisher Scientific Spectra 300 microscope operated at 300 kV. It was conducted with a small convergence semiangle of 110 μrad, a beam current of 1 pA, and an exposure time of 1 ms to achieve the low-dose condition to probe the pristine structure of FAPbI_3_.

## Supporting Information Available

In situ PLM imaging of δ-to-α phase transition in the FAPbI_3_ thin film upon annealing in N_2_ (MP4)
Details of DFT calculations on surface energy; synthesis protocols for δ-FAPbI_3_ thin films; morphology results including AFM height profile, profilometry thickness, and rocking curve, along with an illustration of the PLM mechanism for distinguishing anisotropic and isotropic materials and corresponding confocal PL spectra; and supplementary notes on kinetics analysis using the JMA model and beam effect analysis (PDF)
