# Supplementary material for: Anisotropic δ-to-α Phase Transition in Formamidinium Lead Iodide Thin Films
Source: ACS Nano. 2025 Feb 25;19(9):9225–31. doi: 10.1021/acsnano.5c00037 (PMC11912570; doi:10.1021/acsnano.5c00037)
Supplement: Supplementary file 2 — nn5c00037_si_002.pdf [file nn5c00037_si_002.pdf]

Supplementary Information for the

Anisotropic δ-to-α Phase Transition in Formamidinium Lead Iodide Thin Films

Chen Yang[†], Changsheng Chen[†], Tieyuan Bian[‡], Chao Xu[†], Xiangli Che[†], Dongyang Li[ƻ], Kuan Liang[†], Xuezhe Dong[‡], Jun Yin[‡], Gang Li[*ƻ], Ye Zhu[*†]

[†] Department of Applied Physics, Research Institute for Smart Energy, The Hong Kong Polytechnic University, Hung Hom, Kowloon, Hong Kong, China

Email: yezhu@polyu.edu.hk

[‡] Department of Applied Physics, The Hong Kong Polytechnic University, Hung Hom, Kowloon, Hong Kong, China

[ƻ] Department of Electrical and Electronic Engineering, Research Institute for Smart Energy, Photonic Research Institute, The Hong Kong Polytechnic University, Hung Hom, Kowloon, Hong Kong, China

Email: gang.w.li@polyu.edu.hk

Outline

I. Calculation details

II. Supplementary Figures

1. **Figure S1.**
2. **Figure S2.**
3. **Figure S3.**
4. **Figure S4.**

III. Supplementary Notes

1. **Note S1:** Kinetic analysis using JMA model
2. **Note S2:** Beam effect analysis on stacking stability of $\{1\bar{2}10\}$ and $\{0002\}$

I. Calculation details

The DFT calculations were performed using the projector-augmented wave (PAW) method, as implemented in the Vienna Ab initio Simulation Package (VASP).[1, 2] The generalized gradient approximation (GGA) in conjunction with the Perdew-Burke-Ernzerhof (PBE) exchange correlation functional was employed. The van der Waals (vdW) interactions were incorporated during structural optimization through the zero-damping method of Grimme (DFT-D3).[3] For the optimization of the crystal structures of the α- and δ-phase $FAPbI_3$ bulk and slabs, a Γ-centered grid with a *k*-point density of 0.25 $Å^{-1}$ and Gaussian smearing with a width of 0.05 eV were used. The energy cutoffs of the wavefunctions were set at 500 eV. To avoid interactions between periodic layers, the slab models were placed in the middle of crystals and were separated by vacuum layers of 10 Å at both the top and bottom. The structural optimizations were considered convergent when the force on each atom was less than 0.01 eV/Å.

The $FAPbI_3$ slabs were constructed to simulate the crystalline surfaces along the $\{001\}$, $\{110\}$, $\{111\}$ and $\{210\}$ crystallographic orientation for the α-$FAPbI_3$, and along the $\{0002\}$, $\{10\bar{1}0\}$ and $\{1\bar{2}10\}$ crystallographic orientation for the δ-$FAPbI_3$. The surface energy (σ) was calculated by the following equation:

$$\sigma = \frac{1}{2A}\{\left(E_{slab}^{unrel} - nE_{bulk}\right) + (E_{slab}^{rel} - E_{slab}^{unrel})\}$$

where *A* is the surface area; E_{slab}^{unrel} and E_{slab}^{rel} are the energies of the unrelaxed and relaxed slabs, respectively; E_{bulk} is the energy of the bulk α-$FAPbI_3$ and δ-$FAPbI_3$ structure; and *n* is the exact number of $FAPbI_3$ units in slab models.

II. Supplementary figures

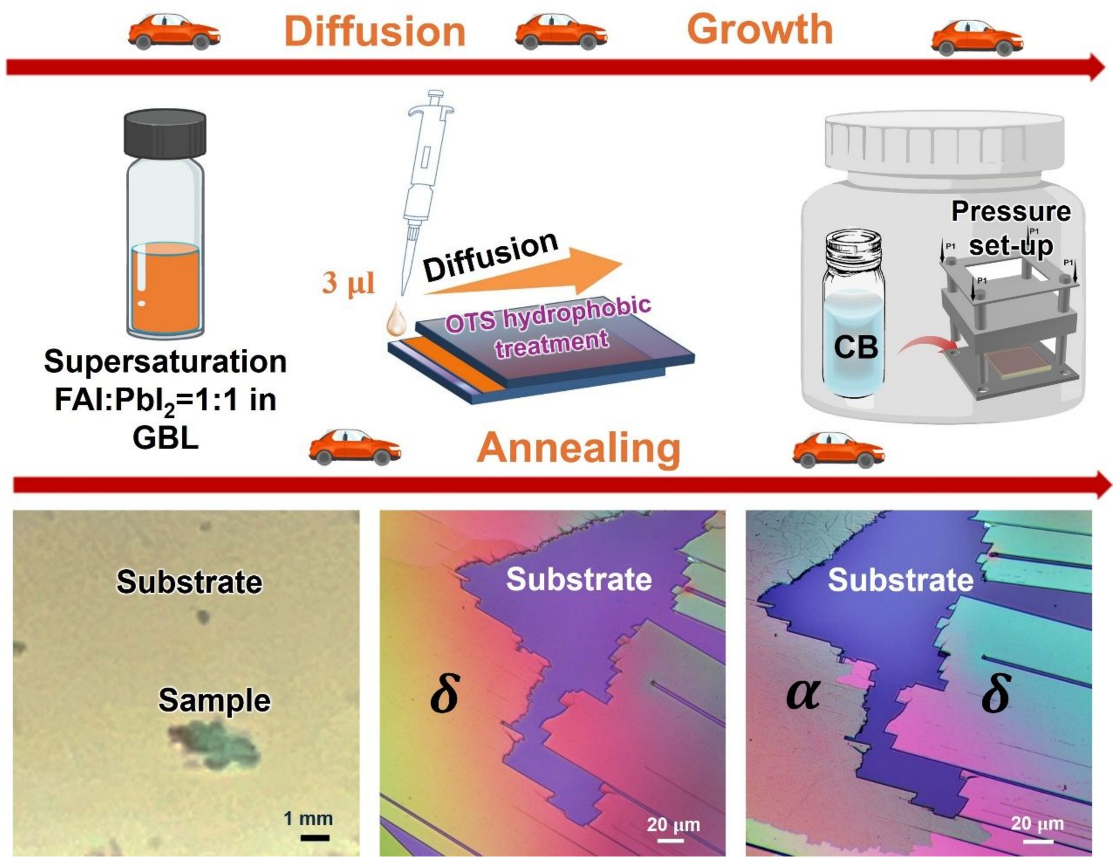

Figure S1. Synthesis protocol of epitaxial δ-$FAPbI_3$ thin films using a combination of space-confined and antisolvent methods, and optical images of a film before and after annealing.

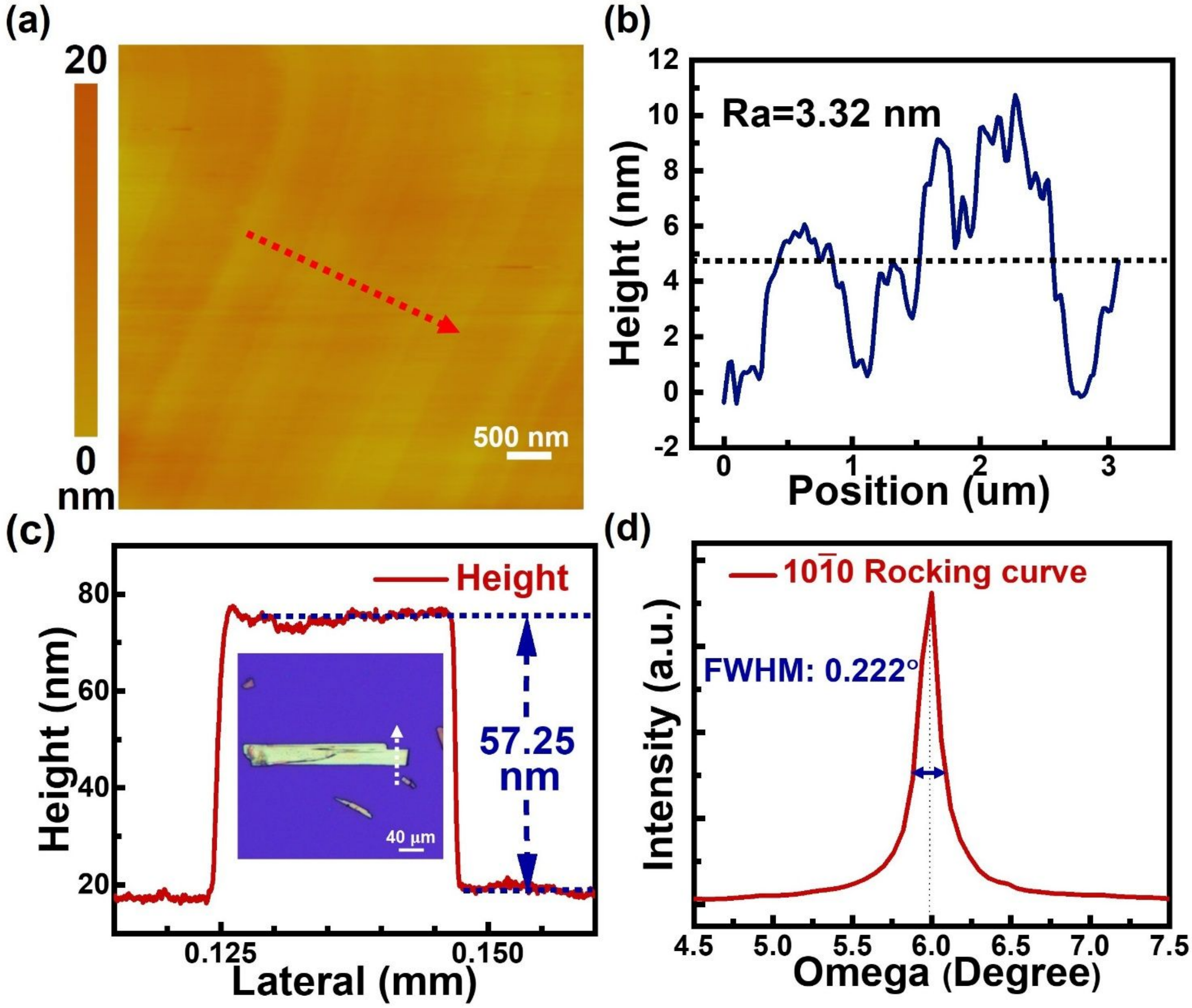

Figure S2. (a) AFM height map of a δ-$FAPbI_3$ thin film. (b) Height profile along the red arrow in **(a)**, showing a surface roughness (Ra) of 3.32 nm. (c) Profilometry thickness measurement, with the inset optical image indicating the scan direction over the sample. (d) Rocking curve measurement on $10\bar{1}0$ diffraction with full width at half maximum (FWHM) ~ 0.222°.

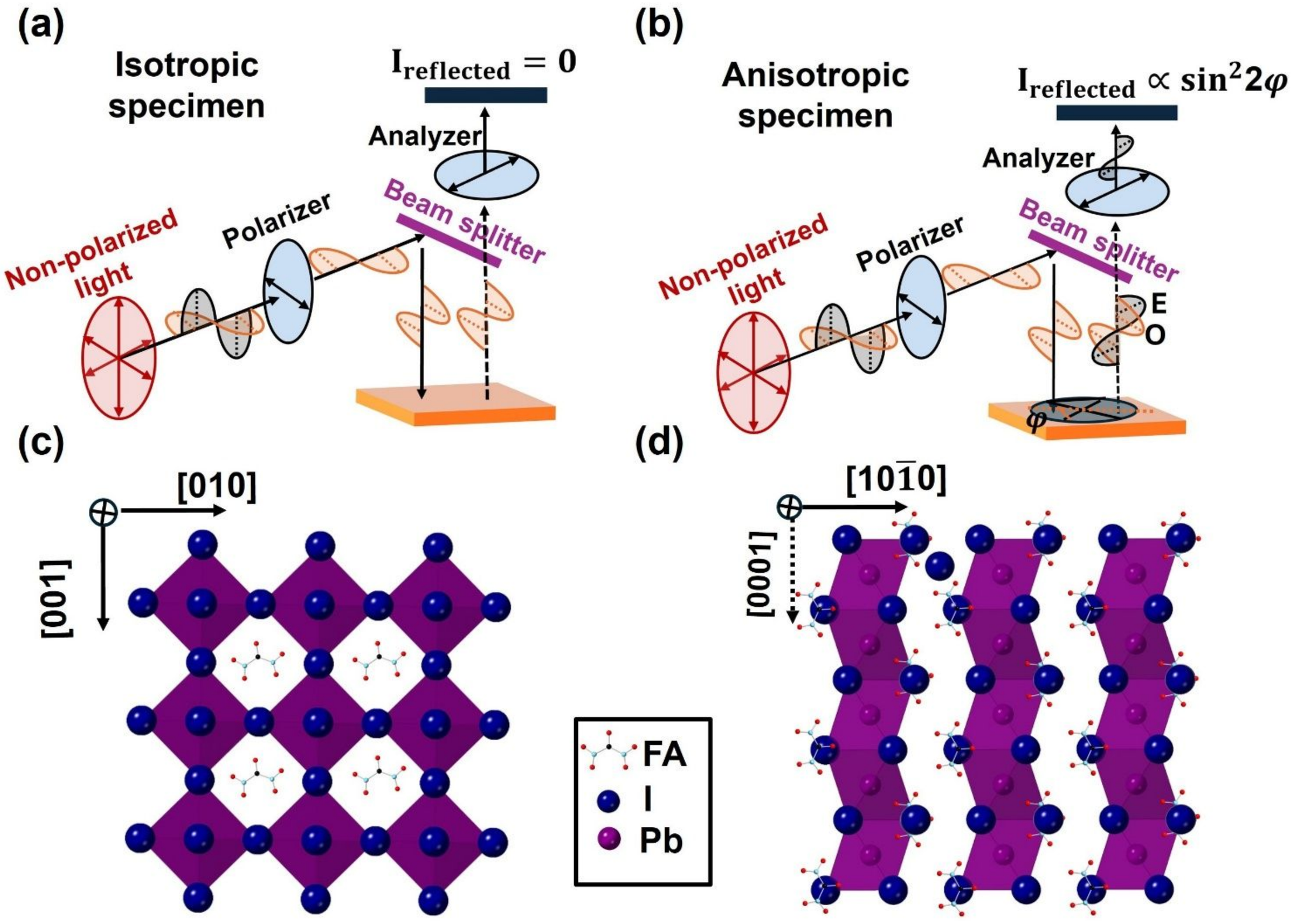

Figure S3. Illustration of the PLM mechanism on distinguish δ/α FAPbI_3:[4] (a,b) Schematics showing (a) unchanged light polarization direction from an isotropic specimen, resulting in extinction in PLM, and (b) polarized light separation into ordinary (O) and extraordinary (E) rays from an anisotropic material, showing no extinction in PLM. (c,d) Atomic structure of (c) isotropic α-FAPbI_3 and (d) anisotropic δ-FAPbI_3 with a single optical axis along the <0001> direction.

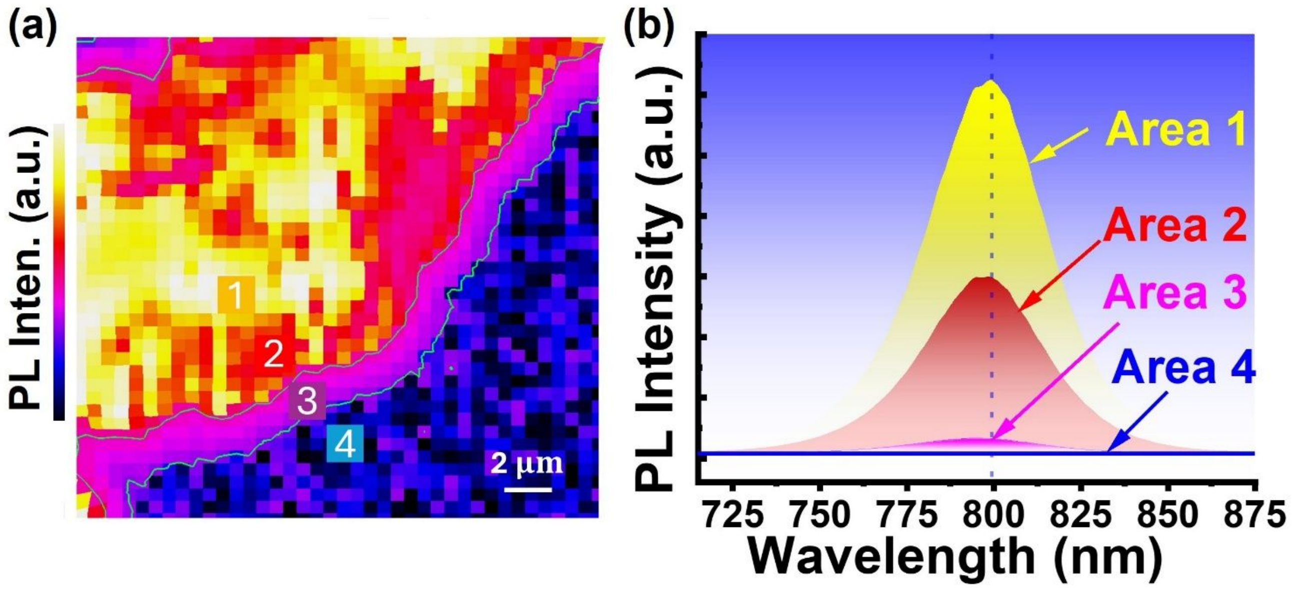

Figure S4. (a) Confocal PL map with the selected area highlighted. (b) Corresponding PL spectra for the indicated areas in **(a)**.

III. Supplementary Notes

Note S1: Kinetics analysis using JMA model

Analysis of kinetics of solid-state phase transformation relies on the precise determination of reaction fraction.[5] Here transformation fraction x_t is defined as the over-time phase transition area of α-$FAPbI_3$ (A_t as measured from *in situ* heating PLM) normalized by the total observation area $x_t = A_t/A_{total}$. Such 2D area fraction can represent 3D volume fraction if the transformation happens thoroughly along the thickness that is <100 nm. This has been confirmed by 4D-STEM as shown in Figure S5: with the scan step ~94 nm, a sharp transition front can still be identified, with overlapped diffraction from the two phases only observed at one position. With much lower resolution in PLM images, it is thus reasonable to assume a sharp transition front with complete phase transition through the whole thickness. Single nucleation is selected in all experiments, so the kinetics reflect the crystal growth process solely without considering the nucleation rates.

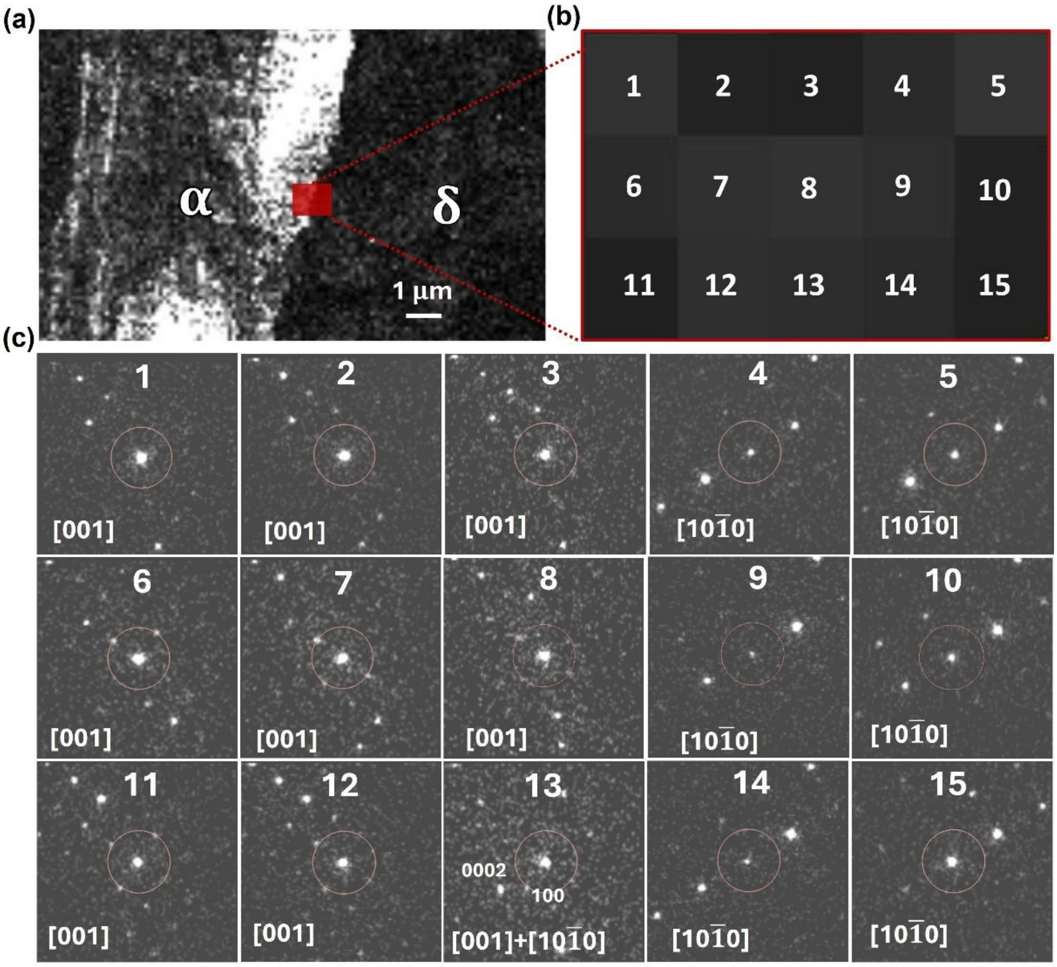

Figure S5. (a) Reconstructed dark-field image using 001 diffraction showing the δ-to-α transition front

in FAPbI$_3$. (b) Selected 5x3 scan region across the transition front. (c) Corresponding electron diffraction patterns at each scan position, where the radius of the circles represents 1/d_{100}.

In this work, we employ the isothermal derivation of JMA equation performed by Mittemeijer et al: [6]

$$ln(t_{x2} - t_{x1}) = \frac{E_a}{RT} - lnk_0 + ln\,(\beta_{x2} - \beta_{x1})$$

where t_{xn} represents the time at which the transformation fraction x_n is achieved, and β_{xt} is a state property corresponding to the transformation fraction x_t in this study. Other parameters are the same as the equation (1) in the main text. This equation allows us to derive the activation energies E_a using the plot in Figure 2c in the main text. In particular, by separating and extracting the transformation rates along the <0001> and <1$\bar{2}$10> directions, we can derive the anisotropic E_a values along the two directions. It's worth noting that the measured transformation rate along <0001> based on our observation (~3.18 μm/s) is very close to the reported results from Lai et al (~3 μm/s).[7]

Figure S6 plots the transformation fraction data along with the derivation of the growth exponent n from the JMA equation (1): $ln(-\,ln(1-x)) = ln\left(k_0 exp\left(\frac{-E_a}{RT}\right)\right) + nln(t)$. It yields n ~2.09 at 150°C and ~2.12 at 170°C respectively. In contrast to n = 3 for typical isotropic phase transformations, n ~ 2 here is attributed to the slow transition rate along <1$\bar{2}$10>, which leads to 2D growth along the other two orthogonal directions (<0002> and <10$\bar{1}$0>). It unambiguously demonstrates the anisotropic phase transition in our epitaxial FAPbI$_3$ thin films.[8]

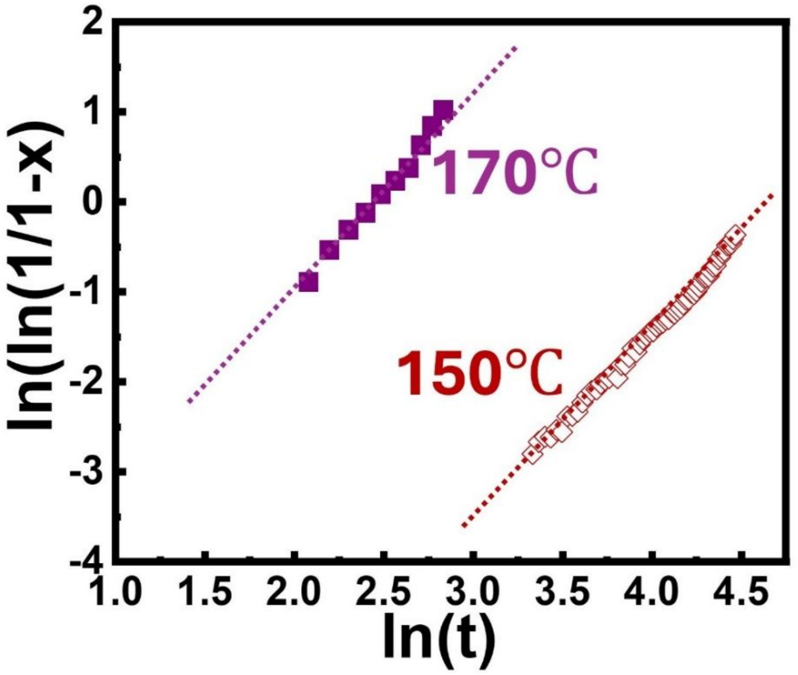

Figure S6. Estimation of the growth exponent n from the plot of the δ-to-α phase transition at 150 °C and 170 °C.

Note S2: Beam effect analysis on stacking stability of $\{1\bar{2}10\}$ and $\{0002\}$

Beam effect on δ-$FAPbI_3$ films was examined with continuous exposure of electron beam until dose accumulates to ~11 e/Å². With increasing electron dose, 0002 and $1\bar{2}10$ diffraction signals exhibit distinct behavior. As shown by the intensity profiles in Figure S7a, intensity of 0002 diffraction decreases rapidly to 82% at ~11 e/Å², while $1\bar{2}10$ diffraction shows a steady response without significant changes. Notably, at this electron dose, no additional diffraction spots or positional shifts of existing diffraction was observed. This is consistent with the work done by Yang *et al.* who derived a critical dose of approximately 12.6 e/Å² in their beam effect study on α-$FAPbI_3$.[9]

Figure S7b plots the relative peak width of 0002 and $1\bar{2}10$, showing the elongation of 0002 diffraction but not for $1\bar{2}10$. It is also inline with the observation of Yang *et al*., who identified more stacking faults induced by the electron dose ranging from 2.1 to 16.8 e/Å² along <111> in α-$FAPbI_3$, which corresponds to <0001> in our δ-$FAPbI_3$.[9] Our observation clearly indicates the relatively unstable $\{0002\}$ stacking compared with $\{1\bar{2}10\}$ stacking, which may promote $FAPbI_3$ phase transition preferentially along <0001>.

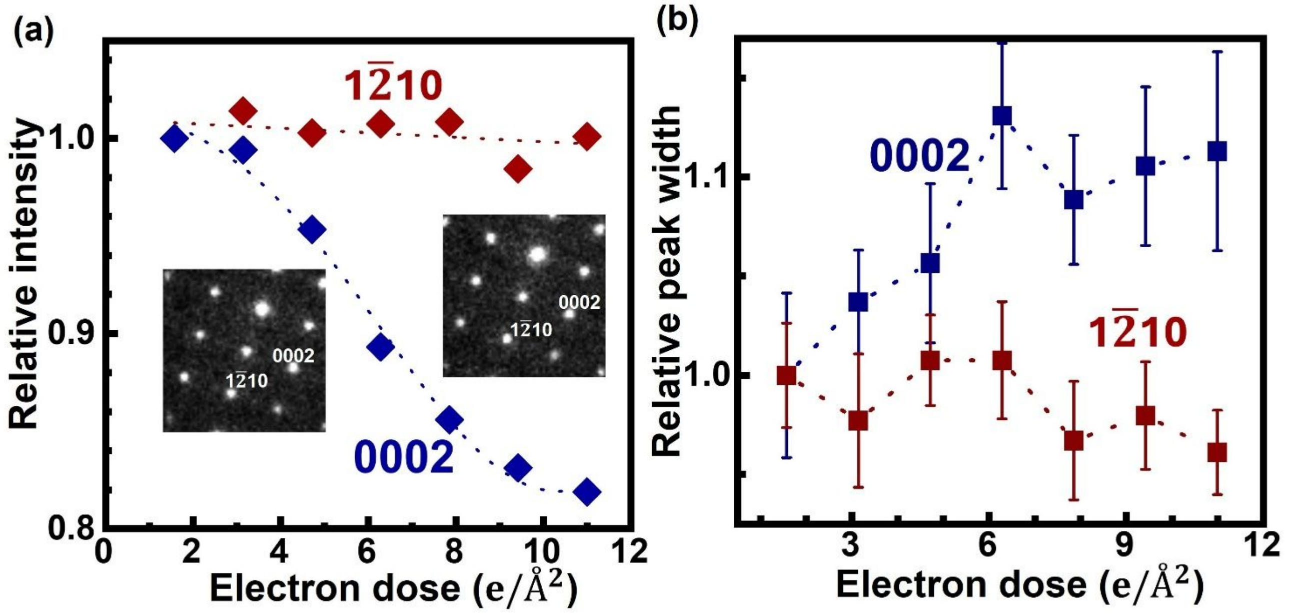

Figure S7. (a) Normalized intensity of $1\bar{2}10$ and 0002 diffraction as a function of accumulated electron dose. The inset shows the diffraction patterns captured at the initial (left) and final (right) stages of beam exposure. (b) Measured 0002 and $1\bar{2}10$ peak width along <0001> and <$1\bar{2}10$> respectively.

REFERENCES

(1) Kresse, G.; Furthmüller, J. Efficiency of ab-initio total energy calculations for metals and semiconductors using a plane-wave basis set. *Computational materials science* **1996**, *6* (1), 15-50.

(2) Kresse, G.; Hafner, J. Ab initio molecular dynamics for liquid metals. *Physical review B* **1993**, *47* (1), 558.

(3) Brooks, K. III i I. *Statistics* **2000**, *2002* (2004), 2001.

(4) Xu, C.; Mao, J.; Guo, X.; Yan, S.; Chen, Y.; Lo, T. W.; Chen, C.; Lei, D.; Luo, X.; Hao, J. Two-dimensional ferroelasticity in van der Waals β'-In2Se3. *Nature Communications* **2021**, *12* (1), 3665.

(5) Moore, D. T.; Sai, H.; Tan, K. W.; Smilgies, D.-M.; Zhang, W.; Snaith, H. J.; Wiesner, U.; Estroff, L. A. Crystallization kinetics of organic–inorganic trihalide perovskites and the role of the lead anion in crystal growth. *Journal of the American Chemical Society* **2015**, *137* (6), 2350-2358.

(6) Mittemeijer, E. Analysis of the kinetics of phase transformations. *Journal of Materials science* **1992**, *27*, 3977-3987.

(7) Lai, M.; Lei, T.; Zhang, Y.; Jin, J.; Steele, J. A.; Yang, P. Phase transition dynamics in one-dimensional halide perovskite crystals. *MRS Bulletin* **2021**, *46*, 310-316.

(8) Readey, D. W. *Kinetics in materials science and engineering*; CRC Press, 2017.

(9) Yang, C. Q.; Zhi, R.; Rothmann, M. U.; Xu, Y. Y.; Li, L. Q.; Hu, Z. Y.; Pang, S.; Cheng, Y. B.; Van Tendeloo, G.; Li, W. Unveiling the intrinsic structure and intragrain defects of organic–inorganic hybrid perovskites by ultralow dose transmission electron microscopy. *Advanced Materials* **2023**, *35* (17), 2211207.
